# Knowledge and management of female genital schistosomiasis in sub-Saharan Africa: A scoping review protocol

**DOI:** 10.4102/sajid.v39i1.553

**Published:** 2024-06-30

**Authors:** Comfort D. Tetteh, Jabulani R. Ncayiyana, Sizwe E. Makhunga, Alfred K. Manyeh, Emmanuel A. Asiamah, Themba G. Ginindza

**Affiliations:** 1 Discipline of Public Health Medicine, School of Nursing and Public Health, University of KwaZulu-Natal, Durban, South Africa; 2 Ghana Health Service, Ayawaso East Municipal Health Directorate, Accra, Ghana; 3 Institute of Health Research, School of Allied Health Science, University of Health Allied Sciences (UHAS), Ho, Ghana; 4 Cancer and Infectious Disease Epidemiology Research Unit (CIDERU), College of Health Sciences, University of KwaZulu-Natal, Durban, South Africa; 5 Department of Medical Laboratory Sciences, School of Allied Health Science, University of Health and Allied Sciences (UHAS), Ho, Ghana

**Keywords:** knowledge, management, female genital schistosomiasis, sub-Saharan Africa, women of reproductive age

## Abstract

**Background:**

Approximately 20 to 120 million women of reproductive age worldwide are thought to be affected by female genital schistosomiasis (FGS). It is a preventable manifestation of schistosomiasis in adolescent girls and women, which remains underreported, underdiagnosed, or misdiagnosed, and largely untreated.

**Objective:**

This study aimed to map evidence on the knowledge and management of FGS from 1950 to 2022 in sub-Saharan Africa.

**Method:**

The Arksey and O’Malley and Levac et al. framework suggestions and a guideline from Joanna Briggs Institute will be employed. Search for literature will be in PubMed, Scopus, Cochrane, Web of Science, MEDLINE via PubMed, and Google Scholar from 1950 to 2022 for useful published research articles using key phrases or search terms and grey literature with limitations for studies conducted in sub-Saharan Africa. Two reviewers will screen the articles. Kappa coefficients by Cohen statistics will be computed for inter-screener agreement, and the selected articles will be evaluated using Mixed Method Appraisal Tool (MMAT).

**Results:**

The researchers will map and explore the evidence of the knowledge and management of FGS in the subregion. The years of publications, countries of study, and settings will be reported, and the identified research gaps will be reported.

**Conclusion:**

The researchers anticipate that this study will determine and map the evidence on the knowledge and management of FGS in sub-Saharan Africa; identify knowledge and management gaps, and direct future research.

**Contribution:**

This study will add to the literature on FGS and direct future research regarding the knowledge and management of FGS.

## Introduction

Schistosomiasis is a debilitating tropical disease ranked third after malaria and intestinal helminthiasis in terms of its occurrence.^1,2^ The disease is caused by trematode parasites of the genus *Schistosoma*, which people contract through direct skin contact with infested freshwater during routine activities such as agricultural, domestic, occupational and recreational activities.^1^ Human beings are affected by 6 out of the 24 species. However, of the six species, *S. haematobium, S. mansoni* and *S. japonicum* are the most common species.^3^ Hybrid between animal and human species is increasingly recognised as human pathogens. Whereas intestinal schistosomiasis is caused by *S. mansoni* and *S. japonicum,* urogenital schistosomiasis is caused by *S. haematobium*.^4^ Approximately 240 million infected individuals with 3.3 million disability-adjusted life-years have been documented worldwide, and over 90% of the infected individuals live in sub-Saharan Africa.^2,5,6^ About 105.4 million received praziquantel treatment out of the projected 236.6 million susceptible individuals in schistosomiasis-endemic areas in 2019.^2^

Female genital schistosomiasis (FGS), a clinical expression of *S. haematobium* caused by the implantation of parasitic eggs in adolescent girls and women’s genital tracts, affects approximately 20 to 120 million women and girls globally.^7,8,9,10^ Gyapong and Theobald asserted that this projection might not accurately reflect the global impact of FGS.^9^ Historically, FGS was first reported in 1899 in Egypt; other reports surfaced in the early 20th century, and others have followed into the present century.^11,12,13,14,15^ However, FGS continues to be poorly recognised, misdiagnosed, underreported and often untreated.^7^ Female genital schistosomiasis is often unrecognised early and affects the same populations that are most severely impacted by cervical cancer and human immunodeficiency virus (HIV) globally.^16,17,18,19^ It is an example of the difficulties women and girls encounter as they deal with numerous and interconnected health, sociocultural, environmental and economic issues.^7^ Colposcopy can be used to identify painful vaginal lesions, which include grainy and homogenous yellow sandy patches, rubbery papules and aberrant blood vessels.^13,20,21^ Other symptoms include spotting, often combined with abnormal discharge, which can be stigmatising as dyspareunia (coital pain or general discomfort and pain during sex).^7,8,22^ These symptoms are synonymous with sexually transmitted infection (STI); thus, FGS is rarely considered among women who report to health facilities with such presentations, even in schistosomiasis-endemic communities.^9,23^ Female genital schistosomiasis has often been misdiagnosed as STI because of its syndromic presentation and inadequate knowledge of the disease among healthcare workers.^23,24,25^

Female genital schistosomiasis damages the reproductive system, resulting in infertility, ectopic pregnancy, miscarriages, premature babies, small or gestation-age babies and maternal death among other clinical consequences.^22,26,27,28^ The disease raises the risk of developing cervical cancer^29,30,31^ and HIV by three times,^17,19,32^ which is biologically plausible. There is a dearth of literature and information on stigma and discrimination connected to FGS. The pain that FGS causes among women of reproductive age is avoidable by diagnosing them at an early age and providing them with frequent praziquantel medication throughout their lifetime.^22^ Another challenge with FGS is how to diagnose it. Although the World Health Organization (WHO) FGS pocket atlas simplifies FGS diagnosis, tools and skills, for example, colposcope devices are unavailable at the primary healthcare levels where most cases are usually seen.^20,33^

The change of the name of the disease from urinary schistosomiasis to urogenital schistosomiasis by WHO was to increase and improve awareness and emphasise the effect on the genital tract.^34^ Despite these efforts, studies have reported inadequate knowledge and skills to manage the disease by health professionals among community members.^24,35,36,37^ Because of lack of awareness among affected communities and the insufficient expertise and understanding among medical professionals, FGS is an underreported, misdiagnosed and untreated medical condition.^35,38,39^ The FGS menace cannot be adequately addressed without adequately equipping different cadres of healthcare workers with the knowledge and tools to diagnose and manage the disease.^24,25,40,41^ Also, prevention and control of FGS are possible through awareness creation that will enhance early reporting, detection, treatment and chemoprophylaxis from an early age and continue throughout life.^42,43^ There is an urgent need to improve the knowledge base of the populace and build health workers’ competence in early detection, diagnosis and FGS management in efforts that will reduce the burden of FGS in lower- and middle-income countries.^24^

Thus, this study seeks to map the evidence on the knowledge and management of FGS to identify the gaps in the literature. The researchers also expect to identify knowledge gaps in the study findings that will inform future studies to advance the understanding of and treatment of FGS in sub-Saharan Africa.

## Research methods and design

The study will review publications and grey literature on FGS from 1950 to 2022 in sub-Saharan Africa to map the evidence of knowledge and management among young girls and women. This study is part of a multi-phase research to improve Ghana’s FGS knowledge, diagnosis, treatment and management.

### Research design

A scoping review on both published and grey literature using the proposed framework by Arksey and O’Malley^44^ and Levac et al.^45^ and PRISMA Extension for Scoping Reviews (PRISMA-ScR) will guide the reporting of the screening (see Table 1-A2). The framework steps are (1) identifying the research question, (2) identifying relevant studies, (3) study selection, (4) charting the data, (5) collating, summarising and reporting the results and (6) quality appraisal.^44,45^ Because this research is nested in a multi-phase study that already incorporates stakeholders’ input, the researchers will leave out the consultation phase. Two seasoned screeners with expertise in scoping review will carry out the above activities, and any inconsistencies in their responses will be resolved by a neutral screener.

### Identify research question

The eligibility for this study was established using the Population, Concept and Context (PCC) mnemonic, outlined in Table 1.^46^

**TABLE 1:** Description of the eligibility of the research question using the Population, Concept and Context framework.

Criteria	Determinants
Population	Women 15–49 years of age
Concept	Knowledge, diagnosis and management of female genital schistosomiasis
Context	SSA

*Source:* The Joanna Briggs Institute. The Joanna Briggs Institute Reviewers’ Manual 2015: Methodology for JBI Scoping Review. Australia: The Joanna Briggs Institute; 2015

SSA, sub-Saharan Africa.

#### Research question

What are the gaps in the literature on knowledge and management of FGS in sub-Saharan Africa?

#### Sub-questions

- What is the burden of FGS among women of reproductive age in sub-Saharan Africa?
- What is the level of knowledge and awareness affected communities have about FGS in sub-Saharan Africa?
- What interventions are implemented to improve case detection and management of FGS in sub-Saharan Africa?

### Identify relevant studies

The researchers will conduct an all-inclusive search in PubMed and an academic search via EBSCOhost, Scopus, Web of Science, MEDLINE and Google Scholar between 1950 and 2022 for relevant articles. The researchers will work with a subject librarian at the University of KwaZulu-Natal to build a search strategy that will allow us to find all and/or most relevant publications for the study. Boolean ‘AND’ and ‘OR’ phrases and Medical Subject Heading (MeSH) terms will be introduced in the search. To collect all eligible research papers, the researchers will use the following keywords: FGS, urogenital schistosomiasis, knowledge, diagnosis, treatment and management. Also, the citation lists of each included study will be examined for relevant studies that would help answer this scoping review question. To search for grey literature, websites of international organisations such as WHO and Centers for Disease Control and Prevention (CDC), dissertations and/or theses, conference proceedings and essential government reports reporting on FGS diagnosis and management in sub-Saharan Africa will be conducted. A pilot search to show this scoping review is feasible as shown in Appendix 2, Table 1-A1.

### Study selection

Three phases will be used to screen for potentially eligible studies. In phase one, one researcher will do a thorough search for eligible papers in electronic resources and export them into a new endnotes X20 library prepared for the study. Deletion of duplicates will be agreed upon and done by co-screeners to clean the library. Using the eligibility criteria created using a Google Form, two screeners will independently screen the abstracts in parallel in this phase. Any disagreements about the article screeners select will be settled by talks among the screeners until an agreement is reached. Lastly, full articles will be examined independently by the two screeners. Any inconsistencies in their responses will be addressed by bringing in a third researcher. To evaluate the degree of inter-screener agreement on the articles chosen for the scoping review, the Kappa coefficient of Cohen statistics will be produced.^47^ The same technique will be used to screen all publications collected from the reference lists of included studies. Researchers may contact the authors to request the entire text of an article for screening if it cannot be located or is not available through online databases or seek help via the University of KwaZulu-Natal library.

**FIGURE 1-A2:**
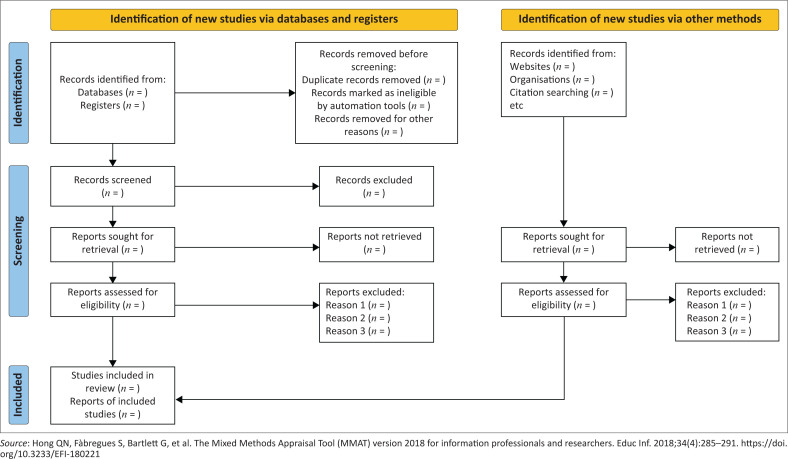
Sample PRISMA 2020 flow diagram for systematic reviews which included searches for databases, registers, and other sources.

The study’s article selection will be reported on using a modified PRISMA-ScR flow diagram, as shown in Appendix 2, Figure 1-A2.

#### Inclusion criteria

Articles inclusion will be based on the following:

- Articles that are written in English between 1950 to 2022.
- Articles reporting on FGS in women of reproductive age.
- Articles reporting on the diagnosis and treatment of FGS.
- Articles reporting on FGS conducted in sub-Saharan Africa.
- Articles that are qualitative, quantitative or product of mixed methods.

#### Exclusion criteria

Article exclusion will be based on the following:

- Articles published outside of sub-Saharan Africa.
- Articles published before 1950.
- Review articles.
- Articles not reporting on the knowledge, diagnosis and treatment of FGS.

### Charting the data

To gather data from the various relevant articles, a data graphing form will be created using Google Forms. Piloting of the form will be done by the researchers to ensure all relevant information is included. To ensure accuracy and ability to gather all eligible data to answer the study question, the form will be revised as applicable. The researchers will continue revising the form throughout this process until all pertinent information from the listed publications has been extracted. The following information will be recorded on the form as follows:

- Author and year of publication.
- Aim of the research.
- Target population studied.
- Research settings (rural or urban).
- Geography (sub-Saharan Africa country conducted).
- The sample size used for the research.
- Age of women (15–49 years).
- Research design used.
- Type of diagnosis (forms of diagnosis).
- Type of management.
- Main findings of the study (diagnosis and management of FGS).
- Other significant findings.
- The target for knowledge assessment.
- Study design used.
- Key findings.
- Conclusions.

### Collating, summarising and reporting the results

NVivo version 12 (Lumivero, Burlington, United States [US])^48^ will be employed to extract the themes from the articles included for the scoping review. Descriptive statistics will be used to present some findings using frequencies and proportions and presented on tables, graphs and maps. To gather relevant information, deductive (such as specific features of the eligible publications) and inductive (such as research findings or outcomes) approaches will be used. This will enable the researchers to analyse all included studies’ thematic content. Utilising the goal and research question, the researchers will offer a narrative summary of the findings that highlight the key ideas from the publications included. The content of our study is the diagnosis and management of FGS, defined as the methods for diagnosing the disease and the means used to manage the disease burden.

### Quality appraisal

The Mixed Method Appraisal Tool (MMAT)^49^ will be used to appraise the articles selected for the study by the two screeners. The quality of evidence will be rated as (1) a percentage score of 50% as low-quality evidence, (2) above 50% but equal to 74% as moderate-quality evidence and (3) 75% will represent high-quality evidence. Through this process, researchers will appraise all study types. The MMAT is available in Appendix 2, Table 2-A2.

## Discussion

Many strategies are being implemented to reduce the burden and the plight of neglected tropical diseases (NTDs) on affected populations worldwide as an effort towards Sustainable Development Goal (SDG) 3 and its targets to promote the well-being of all persons.^50^ These efforts include the planned strategies of WHO NTD Roadmap 2012 to 2020, 2021 to 2030, the Countdown of NTD funded by the UKAID and individual country’s masterplan for NTDs.^51,52,53^ All these efforts are geared towards the control and elimination of NTDs including schistosomiasis.

Despite the implementation of the annual MDA aimed at schistosomiasis control and elimination in endemic areas, the disease burden continues to be high in sub-Saharan Africa^54,55^ with FGS worsening the plight among women of reproductive age.^13,27,56,57,58^ Studies have revealed a multi-disciplinary approach is required to integrate different efforts to eliminate schistosomiasis.^7,34,59^ Lack of awareness about the disease in schistosomiasis-endemic areas and inadequate knowledge among healthcare workers account for the underreporting and underdiagnosis.^24,35,36,37,38^ Any neglected tropical disease can be effectively controlled and prevented if healthcare workers have adequate knowledge, skills and tools to diagnose and manage the condition.^40^ Hence, the implementation of the FGS Accelerated Scale Together (FAST) package to bridge the gap by training different cadres of healthcare workers in some districts.^25^

The study will explore the evidence on the knowledge, diagnosis, treatment and management of the FGS condition among women aged 15–49 in sub-Saharan Africa from 1950 to 2022. The researchers will include evidence on the knowledge, approaches to diagnosing and FGS management. Therefore, the findings and conclusions derived will assist scholars, policymakers and other parties in informing programme guidelines and ensuring efficient healthcare funding distribution. Additionally, this will contribute to increasing the healthcare system’s effectiveness, hence, strengthening interventional and preventive measures for FGS. This study will map evidence of sub-Saharan Africa because of the high burden of the disease in the region,^7^ and findings are more likely to be applied to Ghana which is in the subregion. The findings of this review will reveal the gaps in the knowledge, diagnosis and management of FGS, and inform future research in this region.

### Strengths and limitations

- This study will use primary studies to map the evidence on FGS knowledge, diagnosis and treatment in sub-Saharan Africa.
- This study will draw on both studies that have undergone peer review and unpublished research and reports to address the study’s aim.
- Only articles published in sub-Saharan Africa between 1950 and 2022 will be included.
- Quality appraisal of all articles included will be performed for this review.

## Conclusion

The researchers anticipate this study will identify and map the evidence on the knowledge and management of FGS in sub-Saharan Africa. This study will contribute to the literature and direct future research regarding the knowledge and management of FGS.

## Appendix 1

### Preferred reporting items for systematic reviews and meta-analyses extension for scoping reviews (PRISMA-ScR) checklist

**TABLE 1-A1:** Preferred reporting items for systematic reviews and meta-analyses extension for scoping reviews checklist showing pages for itemised sections.

Section	Item	PRISMA-ScR checklist item	Reported on page #
**Title**			
Title	1	Identify the report as a scoping review.	1
**Abstract**			
Structured summary	2	Provide a structured summary that includes (as applicable): background, objectives, eligibility criteria, sources of evidence, charting methods, results, and conclusions that relate to the review questions and objectives.	1
**Introduction**			
Rationale	3	Describe the rationale for the review in the context of what is already known. Explain why the review questions/objectives lend themselves to a scoping review approach.	3
Objectives	4	Provide an explicit statement of the questions and objectives being addressed with reference to their key elements (e.g. population or participants, concepts, and context) or other relevant key elements used to conceptualize the review questions and/or objectives.	4
**Methods**			
Protocol and registration	5	Indicate whether a review protocol exists; state if and where it can be accessed (e.g. a Web address); and if available, provide registration information, including the registration number.	10
Eligibility criteria	6	Specify characteristics of the sources of evidence used as eligibility criteria (e.g. years considered, language, and publication status), and provide a rationale.	7
Information sources	7	Describe all information sources in the search (e.g. databases with dates of coverage and contact with authors to identify additional sources), as well as the date the most recent search was executed.	7
Search	8	Present the full electronic search strategy for at least one database, including any limits used, such that it could be repeated.	7
Selection of sources of evidence	9	State the process for selecting sources of evidence (i.e. screening and eligibility) included in the scoping review.	7
Data charting process	10	Describe the methods of charting data from the included sources of evidence (e.g. calibrated forms or forms that have been tested by the team before their use, and whether data charting was done independently or in duplicate) and any processes for obtaining and confirming data from investigators.	7
Data items	11	List and define all variables for which data were sought and any assumptions and simplifications made.	8
Critical appraisal of individual sources of evidence	12	If done, provide a rationale for conducting a critical appraisal of included sources of evidence; describe the methods used and how this information was used in any data synthesis (if appropriate).	8
Synthesis of results	13	Describe the methods of handling and summarising the data that were charted.	8
**Results**			
Selection of sources of evidence	14	Give numbers of sources of evidence screened, assessed for eligibility, and included in the review, with reasons for exclusions at each stage, ideally using a flow diagram.	8
Characteristics of sources of evidence	15	For each source of evidence, present characteristics for which data were charted and provide the citations.	8
Critical appraisal within sources of evidence	16	If done, present data on critical appraisal of included sources of evidence (see item 12).	8
Results of individual sources of evidence	17	For each included source of evidence, present the relevant data that were charted that relate to the review questions and objectives.	8
Synthesis of results	18	Summarise and/or present the charting results as they relate to the review questions and objectives.	8
**Discussion**			
Summary of evidence	19	Summarise the main results (including an overview of concepts, themes, and types of evidence available), link to the review questions and objectives, and consider the relevance to key groups.	9
Limitations	20	Discuss the limitations of the scoping review process.	10
Conclusions	21	Provide a general interpretation of the results with respect to the review questions and objectives and potential implications and/or next steps.	10
**Funding**			
Funding	22	Describe sources of funding for the included sources of evidence, as well as sources of funding for the scoping review. Describe the role of the funders of the scoping review.	10

*Source:* The Joanna Briggs Institute. The Joanna Briggs Institute Reviewers’ Manual 2015: Methodology for JBI Scoping Review. Australia: The Joanna Briggs Institute; 2015

## Appendix 2

### Search strategy using Boolean & MeSH terms

**TABLE 1-A2:** Pilot searches on the database to indicate the feasibility of conducting the study.

Database	Search terms	Date searched	Number found
PubMed	(“Schistosomiasis haematobium/diagnosis”[MeSH] OR “Schistosomiasis haematobium/diagnostic imaging”[Mesh] OR “Schistosomiasis haematobium/prevention and control”[Mesh] OR “Schistosomiasis haematobium/therapy”[Mesh])	27/05/2022	471 (2010–2022)
Cochrane	Female genital schistosomiasis, urogenital schistosomiasis knowledge, diagnosis, management	27/05/2022	4 (2010–2022)
Google scholar	urogenital schistosomiasis and female genital schistosomiasis diagnosis treatment knowledge	01/12/2022	8380 (1990–2022)

**TABLE 2-A2:** The mixed method appraisal tool for quality appraisal of the female genital schistosomiasis scoping review.

Question number	Q1	Q2	Q3	Q4	Q5	Q6	Q7	Q8	Q9	Q10	Q11	Q12	Q13	Q14	Q15	Q16	Q17	Q18	Q19	Q20	Q21	Q22
**Qualitative studies**																						
	-	-	-	-	-	-	-	-	-	-	-	-	-	-	-	-	-	-	-	-	-	-
	-	-	-	-	-	-	-	-	-	-	-	-	-	-	-	-	-	-	-	-	-	-
	-	-	-	-	-	-	-	-	-	-	-	-	-	-	-	-	-	-	-	-	-	-
**Quantitative studies**																						
	-	-	-	-	-	-	-	-	-	-	-	-	-	-	-	-	-	-	-	-	-	-
	-	-	-	-	-	-	-	-	-	-	-	-	-	-	-	-	-	-	-	-	-	-
	-	-	-	-	-	-	-	-	-	-	-	-	-	-	-	-	-	-	-	-	-	-
	-	-	-	-	-	-	-	-	-	-	-	-	-	-	-	-	-	-	-	-	-	-
	-	-	-	-	-	-	-	-	-	-	-	-	-	-	-	-	-	-	-	-	-	-
**Mixed methods studies**																						
	-	-	-	-	-	-	-	-	-	-	-	-	-	-	-	-	-	-	-	-	-	-
	-	-	-	-	-	-	-	-	-	-	-	-	-	-	-	-	-	-	-	-	-	-
	-	-	-	-	-	-	-	-	-	-	-	-	-	-	-	-	-	-	-	-	-	-
	-	-	-	-	-	-	-	-	-	-	-	-	-	-	-	-	-	-	-	-	-	-

*Source:* Hong QN, Fàbregues S, Bartlett G, et al. The Mixed Methods Appraisal Tool (MMAT) version 2018 for information professionals and researchers. Educ Inf. 2018;34(4):285–291. https://doi.org/10.3233/EFI-180221

Screening questions (for all types)

- Q1: Are there clear research questions?
- Q2: Do the collected data allow us to address the research questions?

Qualitative

- Q3: Is the qualitative approach appropriate to answer the research question?
- Q4: Are the qualitative data collection methods adequate to address the research question?
- Q5: Are the findings adequately derived from the data?
- Q6: Is the interpretation of results sufficiently substantiated by data?
- Q7: Is there coherence between qualitative data sources, collection, analysis, and interpretation?

Quantitative randomised controlled trials

- Q8: Is randomisation appropriately performed?
- Q9: Are the groups comparable at baseline?
- Q10: Are there complete outcome data?
- Q11: Are outcome assessors blinded to the intervention provided?
- Q12: Did the participants adhere to the assigned intervention?

Quantitative

- Q13: Is the sampling strategy relevant to address the research question?
- Q14: Is the sample representative of the target population?
- Q15: Are the measurements appropriate?
- Q16: Is the risk of nonresponse bias low?
- Q17: Is the statistical analysis appropriate to answer the research question?

Mixed methods

- Q18: Is there an adequate rationale for using a mixed methods design to address the research question?
- Q19: Are the different components of the study effectively integrated to answer the research question?
- Q20: Are the outputs of the integration of qualitative and quantitative components adequately interpreted?
- Q21: Are divergences and inconsistencies between quantitative and qualitative results adequately addressed?
- Q22: Do the different components of the study adhere to the quality criteria of each tradition of the methods involved?
